# Targeting adenovirus gene delivery to activated tumour-associated vasculature via endothelial selectins

**DOI:** 10.1016/j.jconrel.2010.10.011

**Published:** 2011-03-10

**Authors:** Houria Bachtarzi, Mark Stevenson, Vladimir Šubr, Karel Ulbrich, Leonard W. Seymour, Kerry D. Fisher

**Affiliations:** a Department of Clinical Pharmacology, University of Oxford, Old Road Campus, Headington, Oxford, OX3 7DQ, UK; b Institute of Macromolecular Chemistry, Academy of Sciences of the Czech Republic, Heyrovsky Sq. 2, 162 06 Prague 6, Czech Republic

**Keywords:** E-selectin, pHPMA, Adenovirus, Vascular targeting, Cancer

## Abstract

Clinical experience with adenovirus vectors has highlighted the need for improved delivery and targeting. Tumour-associated endothelium offers an additional mechanism for enhanced viral uptake into tumours which is accessible for systemic gene delivery. Building on expertise in using polymer ‘stealthed’ viruses for targeting *in vivo*, adenovirus expressing luciferase (Adluc) was coated with an amino-reactive polymer based on poly [N-(2-hydroxypropyl) methacrylamide] to ablate normal infection pathways. Direct linkage of a monoclonal antibody against E-selectin (MHES) demonstrated E-selectin-specific transduction of tumour necrosis factor-α (TNF-α)-activated endothelial cells. A two-component targeting system using protein G was developed, to provide optimal antibody orientation. We report an enhancement in transduction of TNF-α-activated endothelium *in vitro* and *ex vivo* in a human umbilical vein cord model using the MHES antibody. Similarly a virus retargeted using a chimeric P-selectin Glycoprotein Ligand-1-Fc fusion (PSGL-1) protein showed better circulation kinetics and significant uptake into HepG2 xenografts following systemic administration in mice, with 36-fold higher genome copies, compared with non-modified virus. Immunohistochemistry staining of tumour sections from mice treated with PSGL-1-retargeted virus showed a co-localisation of firefly luciferase with CD31 suggesting selective endothelial targeting. Employment of optimal viral modification using protein G will enable exploration and comparison of alternative targeting ligands targeting tumour-associated endothelium.

## Graphical Abstract

**Figure:**
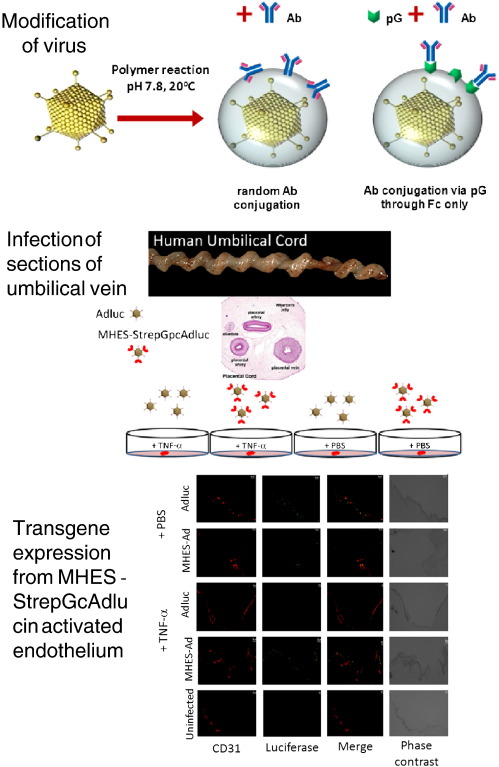

## 1 Introduction

Gene therapy as a concept provides an appealing alternative to small drugs for the treatment of complex diseases like cancer, allowing expression of specific therapeutic proteins rather than being limited to inhibition of tumour-associated targets. In addition, gene therapy offers the advantages of power through amplification of gene products, specificity through the use of tissue-specific promoters and, versatility, delivering agents with different mechanisms of action such as: suicide genes (virus-directed enzyme prodrug therapy) and immuno-modulatory proteins [1]. To date, most cancer gene therapy approaches have targeted tumour cells by local administration protocols, with few having evaluated targeting tumour-associated endothelium [2,3].

Therapeutic strategies that target tumour-associated vasculature have several advantages compared with direct targeting of tumour parenchymal cells. Firstly, the endothelium represents a primary point of contact with blood-borne molecules and is easily accessible from the bloodstream. Secondly, tumour-associated endothelium displays many similar properties regardless of tumour type that distinguish it from normal endothelium, giving rise to a broad class of therapy. Lastly, destruction of a relatively small number of endothelial cells has the potential to deprive many tumour cells of oxygen and nutrients, leading to an avalanche of tumour cell death [4].

Unlike normal endothelium, tumour-associated endothelial cells often display an inflamed phenotype due to activation by cytokines emanating from the tumour including tumour necrosis factor-α (TNF-α) and interferon-γ (INF-γ) [5]. This pro-inflammatory activation promotes faster endothelial proliferation and results in up-regulated levels of cell surface markers. αvβ3 integrins, vascular endothelial growth factor receptors and E-selectin have all been reported to be over-expressed on tumour-associated endothelium [6,7].

Selectins are a family of structurally-related, Ca^2+^-dependent, type-I transmembrane carbohydrate-binding proteins which include: E-selectin (CD62E), P-selectin (CD62P) and L-selectin (CD62L). E-selectin and P-selectin are of particular interest in inflammation and cancer, as they are functionally used by circulating macrophages to home towards the regions of disease [8,9]. Both E- and P-selectins are expressed on activated endothelial lumen, are rapidly internalised via an endosomal pathway, and have successfully been exploited to target tumour-associated vasculature *in vivo* [10,11]. Redirecting viral tropism via endothelial selectins is therefore an attractive option worth further study. Ogawara et al. examined redirecting adenoviral tropism using bifunctional PEG attached to the surface of the virus capsid inhibiting fibre knob/CAR interactions. Subsequent introduction of E-selectin antibodies to functional groups of the PEG molecule allowed retargeting to activated endothelial cells *in vitro* and *in vivo* [12]. Despite the conceptual advantages of targeting therapeutics using viruses to endothelium, this strategy remains fraught with difficulties due to complement [13,14], antibodies [14], blood cell binding [15–17], clotting factor interactions [18,19] and liver capture [20,21]. In an effort to improve virus bloodstream circulation and survivability for a sufficient time to allow vascular targeting, we have used a polymer encapsulation technique based on poly-[N-(2-hydroxypropyl) methacrylamide] (pHPMA) to de-target viruses and protect against unwanted vector–host interactions [17]. Several ligands have previously been successfully incorporated ranging from peptides to monoclonal antibodies [22–24]. One drawback of this approach for using antibodies as targeting ligands is the random nature of antibody orientation on the polymer due to the presence of multiple reactive amino groups. Krasnykh and co-workers have previously shown that genetic modification of the Ad fibre protein incorporating the immunoglobulin (Ig)-binding domain of *Staphylococcus aureus* protein A, created a vector capable of binding targeting ligands incorporating the Fc domain of immunoglobulin. Targeting ligands incorporating CD40 single chain antibodies or CD40L mediated a significant increase in the transduction of CD40-positive target cells [25].

In order to identify receptors and ligands that may be useful for endothelial targeting we chose to use *Streptococcus aureus* protein G (StrepG) as a platform targeting system. The advantage of this approach is that it allows a correctly orientated linkage of Fc-bearing ligands to tropism-ablated polymer coated virus particles. Accordingly in this study we have explored the use of StrepG-based retargeting of polymer coated adenovirus to endothelial selectins *in vitro, ex vivo* and *in vivo*.

## 2 Materials and methods

### 2.1 Cell lines

Human Umbilical Vein Endothelial cells (HUVECs; PromoCell, Heidelberg, Germany) were maintained in EGM^TM^-2-Endothelial Cell Medium-2 (Cambrex Bio Science, Walkersville, USA), supplemented with hydrocortisone, h-FGF-B, VEGF, R3-IGF-1, ascorbic acid, heparin, FBS, hEGF, and GA-1000 (Cambrex Bio Science, Walkersville, USA). Brain endothelioma cell line (bEnd-3; ATCC, Manassas, USA) was grown in Dulbecco's modified Eagle's medium (DMEM) containing 20 mM HEPES, 10% foetal calf serum (FCS) and 2 mM glutamine (PAA laboratories, Yeovil, UK). HepG2 human hepatocyte carcinoma were maintained in Minimal Essential Medium with Earle's salts (EMEM), 2 mM glutamine (PAA Laboratories GmbH, UK), supplemented with 10% FCS, and 0.1 mM non-essential amino-acids. The H18/7 (mouse IgG2a anti-human E-selectin) monoclonal antibody-producing hybridoma (ATCC, Manassas, USA) was grown in RPMI 1640 medium (PAA Laboratories, Yeovil, UK) supplemented with 10% horse serum (Invitrogen, Paisley, UK) with NEAA (PAA laboratories, Yeovil, UK) and 1 mM sodium pyruvate (Sigma-Aldrich, Gillingham, UK).

### 2.2 Antibodies

MHES (mouse IgG2a anti-human E-selectin) monoclonal antibody was purified from the H18/7 culture medium by protein A affinity columns (nProtein A sepharose 4 fast flow, GE Healthcare). The mouse IgG2a isotype control monoclonal antibody was kindly provided by Prof P.E. Thorpe (University of Texas, Southwestern Medical Center, Dallas). RMES (mouse IgG2a anti-rat E-selectin) monoclonal antibody was purchased from BD Pharmingen (Oxford, UK). Recombinant human P-selectin Glycoprotein Ligand-1 (PSGL-1)-/IgG1 Fc fusion protein was purchased from R&D systems (Abingdon, UK). Ad5 fibre was a gift from Dr. R. Carlisle (University of Oxford, UK).

### 2.3 Viruses

E1, E3-deleted Ad5 expressing cytomegalovirus immediate-early (CMV IE) promoter-driven luciferase (Adluc) was grown in HEK293 cells until a cytopathic effect was observed. Virus was released from cells by freeze/thawing, extracted with *N*-butanol and purified by double banding on a CsCl gradient with an intermediate benzonase (Merck Biosciences, Nottingham, UK) step to remove contaminating DNA. The virus was dialysed into 50 mM HEPES/phosphate-buffered saline (PBS), 10% glycerol pH 7.8 (storage buffer) and stored in small aliquots. Particle numbers were estimated using the PicoGreen assay (Molecular Probes, Paisley, UK) assuming 1 μg/ml DNA = 2.7 × 10^10^ particles/ml [26]. Typical particle:infectivity ratios of 10:1 were obtained.

### 2.4 Virus modification

#### 2.4.1 Direct conjugation of antibody

Amino-reactive multivalent hydrophilic polymer based on poly[N-(2-hydroxypropyl)methacrylamide] [pHPMA-gly-gly-TT] [27] supplied by Institute of Macromolecular Chemistry (Prague, Czech Republic) was resuspended in water to make a 100 mg/ml polymer stock solution. Ten μl was then added to 90 μl of virus for 40 min at room temperature (pcAdluc). Free un-reacted polymer was removed from pcAdluc using S400 micro-spin columns (Amersham Biosciences, Buckinghamshire, UK) according to the manufacturer's instructions. Mouse anti-human E-selectin monoclonal antibody (MHES) was conjugated to pcAdluc by adding 0.5 mg/ml antibody to the virus initially for 1 h at room temperature and then overnight at 4 °C.

#### 2.4.2 Indirect conjugation through protein G

pcAdluc was generated by incubating 1 ml of Adluc (1–3 × 10^12^ particles/ml) in storage buffer with the pHPMA-gly-gly-TT polymer at a final concentration of 10 mg/ml for 40 min at room temperature. pcAdluc was purified away from the unincorporated polymer by caesium chloride banding and subsequently mixed with *S. aureus* protein G (StrepG) (Sigma-Aldrich, Gillingham, UK), at a final concentration of 1 mg/ml for 1 h at room temperature before incubation overnight at 4 °C. Protein G-reacted polymer coated virus (StrepGpcAdluc) was purified again by caesium chloride banding to remove unincorporated StrepG proteins. Mouse anti-human E-selectin monoclonal antibody (MHES) (10 μg/ml), rat anti-mouse E-selectin monoclonal antibody (RMES) (1-100 μg/ml) or chimeric P-selectin Glycoprotein Ligand-1 (PSGL-1)-Fc fusion protein (1-50 μg/ml), were linked via their Fc regions to StrepG-modified polymer coated virus through affinity interaction following a 1 h-incubation at room temperature.

### 2.5 Dot blot analysis

The amount of protein G associated with each virus particle was determined by dot blot analysis. A standard curve of protein G ranging from 78 to 5000 pg was prepared by serial dilution and application to a nitrocellulose membrane. Purified StrepGpcAdluc was serially diluted and added to the nitrocellulose membrane (ranging from 1.5 × 10^6^ to 1 × 10^8^ virus particles). The nitrocellulose was then probed with polyclonal anti-protein G antibodies (Abcam, Cambridge, UK) diluted 1:2000, followed by goat anti-rabbit-HRP antibodies (Santa Cruz Biotechnology, Santa Cruz, CA, USA) diluted 1:2500. Signals were visualised using ECL Western blotting detection reagent (GE Healthcare Biosciences, Chalfont St. Giles, UK).

### 2.6 Immunocytochemical analysis

HUVECs were treated with PBS or 100 ng/ml rhTNF-α (R&D Systems Europe Ltd, Abingdon, UK) for 4 h at 37 °C and then analysed for E-selectin expression. Cells were gently scraped and placed into 96 well V-bottom plates. Cells were then incubated with 10 μg/ml MHES antibodies for 40 min at 4 °C, followed by a second incubation (40 min, 4 °C) with a goat anti-mouse antibody, conjugated to R-Phycoerthyrin. Cells were analysed (5 × 10^3^ events counted, approximately 4 × 10^3^ events gated) by flow cytometry at 585 ± 21 nm using a FACSCalibur (Becton Dickinson, Oxford, UK) and CellQuest Pro software (Becton Dickinson, Oxford, UK); the M1 gate was set using the mock (no antibody) control.

### 2.7 Transduction assays

HUVECs or bEnd3 cells were seeded into 96-well plates at 10,000 cells per well 24 h prior to infection and were incubated in triplicate with 1000 virus particles/cell of Adluc, pcAdluc, StrepGpcAdluc or MHES/RMES/PSGL-1-Fc-StrepGpcAdluc in 100 μl infection medium (DMEM, 2% FCS, 2 mM glutamine). After 90 min, the infectious medium was removed and 100 μl normal cell growth medium was added to cells. Transgene expression was measured 24 h later using the bright-Glo^TM^ luciferase assay system (Promega, Southampton, UK) and a Victor 2 plate reader (Perkin Elmer) or a luminometer (LB9507; Berthold Technologies, Bad Wildbad, Germany). Cell lysates were assayed for protein content using the BCA assay (B9643; Sigma-Aldrich, Gillingham, UK).

#### 2.7.1 Competition (blocking) assay

CAR was blocked by incubating cells with 10 μl Ad5 fibre. E-selectin was blocked with 20 μg/ml MHES antibody or isotype control antibody. Cells were blocked at 4 °C for 30 min and without removing antibodies were then infected at 1000 MOI for 1 h at 37 °C. Virus was then removed, fresh media added and transgene expression measured after 24 h.

### 2.8 Infection of umbilical cord *ex vivo*

Human umbilical cords were obtained from healthy females courtesy of the Delivery Suite, Department of Obstetrics and Gynaecology, John Radcliffe Hospital, Oxford. They were stored at 4 °C in sterile PBS and used within 24 h. A fresh human umbilical cord was rinsed with PBS to remove residual blood cells, cut in to small pieces (1–2 cm) with a scalpel, incubated in EGM^TM^-2-Endothelial Cell Medium-2 (2% FCS) and stimulated with or without 50 ng/ml recombinant human TNF-α (rhTNF-α; R&D, Abingdon, UK) for 4 h at 37 °C. The sections of cord were infected with 1 × 10^10^ Adluc or MHES-StrepGpcAdluc particles in serum-depleted growth media. After 90 min, the infectious medium was removed; tissues were washed with PBS and re-incubated in complete HUVEC growth medium for 24 h.

#### 2.8.1 Immunohistochemical staining of frozen umbilical cord sections

Twenty four hours post-virus infection, pieces of umbilical cord were snap frozen in dry ice. Serial Cryostat 10 μm sections were cut, air dried onto microscope polylysine slides (Thermo scientific, Braunschweig, Germany) overnight at 37 °C, and then fixed with Cell-Fixx^TM^ (Thermo Electron Corporation, Cheshire, UK) for 4 h at room temperature. For double-immunofluorescence staining, sections were washed in PBS and blocked with 10% heat-inactivated goat serum (Sigma-Aldrich, Gillingham, UK) containing 0.3% Triton X-100 in PBS for 30 min at room temperature. Primary antibodies: a rabbit anti-luciferase polyclonal and a mouse IgG1 anti-human CD31 monoclonal antibodies (abcam, Cambridge, UK) were diluted 1/1000 and 1/10, respectively in 1% heat-inactivated goat serum containing 0.3% Triton X-100 and incubated on sections for 45 min at room temperature. Sections were then washed in PBS and stained for 45 min at room temperature with a combination of two secondary antibodies: a goat anti-rabbit-Alexa Fluor 488 (Invitrogen, UK), diluted 1/200 in 1% goat serum to detect rabbit anti-luciferase antibody binding and a goat anti-mouse-Alexa Fluor 594 (Invitrogen, UK), diluted 1/200 in 1% goat serum to detect mouse anti-human CD31 antibody binding. Slides were washed in PBS and were then mounted in mounting media containing DAPI and kept in the dark.

#### 2.8.2 Fluorescence microscopy

Sections were imaged using a confocal microscope Nikon Optiphot-2 upright microscope, fitted with 5×, 10×, 20× and 40× objectives, a 60× oil immersion objective and Bio-Rad MRC 1024 with Krypton/Argon lasers, emitting a green light at 488 nm (for Alexa Fluor 488) and a red light at 568 nm (for Alexa Fluor 568) and at 594 nm (for Alexa Fluor 594). Images were processed using LaserSharp 2000 image processing software (Bio-rad).

### 2.9 *In vivo* studies

Female nude mice aged 4–6 weeks (Charles River Laboratories, Kent, UK) were housed and cared for according to Home Office regulations. HepG2 cells (5 × 10^6^) were implanted by subcutaneous injection and animals were monitored on a regular basis until tumours were just palpable and were adequately vascularised.

For kinetics studies, mice were anaesthetised by isofluorane inhalation and virus samples (Adluc or PSGL-1-Fc-StrepGpcAdluc) were injected into the tail vein using a 29-gauge insulin syringe (Becton Dickinson, Oxford, UK). Blood samples were taken from the tail vein at 5, 10, 20 and 30 min after virus injection. A blood volume corresponding to 2.0 ml/mouse was assumed for calculation of virus present in the bloodstream.

For retargeting studies, mice were divided into 4 groups (4 mice each) and given clodronate liposomes (100 μl) followed 24 h later by an intravenous injection 1 × 10^10^ virus particles of Adluc, StrepGpcAdluc, IgG1-StrepGpcAdluc or PSGL-1-Fc-StrepGpcAdluc. After 24 h, the animals were culled; tumours were harvested, frozen in dry ice and cut using a cryostat into 10 μm sections as described above. Sections were incubated with rat anti-mouse CD31 monoclonal antibody to stain for tumour-associated mouse vasculature and anti luciferase antibodies as described above. CD31 was detected using goat anti-rat-Alexa Fluor 568 secondary antibodies. Immunostained sections were analysed by confocal microscopy as described above.

### 2.10 Quantitative-PCR analysis

Quantitative PCR (Q-PCR) was used to detect the presence of adenovirus DNA in extracted DNA samples using an ABI 7000 Sequence Detection System and software. Amplification of an 84 bp fragment of the adenovirus fibre gene was carried out using the forward primer 5′ TGGCTGTTAAAGGCAGTTTGG 3′ and reverse primer 5′ GCACTCCATTTTCGTCAAATCTT 3′ with detection of amplified sequences by a Taqman probe (5′ TCCAATATCTGGAACAGTTCAAGTGCTCATCT 3′), which was labelled at the 5′ end with the FAM fluorophore and at the 3′ end with the TAMRA quencher. Primers and probe were purchased from Sigma Genosys (Pampisford, Cambridgeshire, UK). Reactions were carried out in a total volume of 25 μl in ABI Taqman Master mix (Applied Biosystems, Warrington, Cheshire, UK) containing primers and probes at concentrations of 1.0 and 0.1 μM, respectively. All non-DNA-containing components were UV irradiated for 15 min prior to reactions being set up in a sterile environment. Thermocycling parameters were optimized as 2 min at 50 °C, 15 min at 95 °C followed by 40 cycles of 95 °C (30 s) and 60 °C (2 min). Analysis of data was carried out using ABI software and test samples compared to standards of known viral content based on picogreen analysis. Standard curves were prepared for individual tissue types by spiking serial dilutions of virus into control tissues and extracting DNA (according to manufacturer's instructions) for Q-PCR analysis.

### 2.11 Statistical analysis

The data are expressed as the mean of 3–4 replicates ± standard deviation unless otherwise stated. Significance was evaluated using a one-way ANOVA (analysis of variance) with post-hoc analysis using Tukey's test: ^⁎⁎⁎^p < 0.001, ^⁎⁎^p < 0.01, ^⁎^p < 0.05.

## 3 Results

### 3.1 Transduction efficiency of pHPMA-adenovirus retargeted via E-selectin in non-rhTNF-α- versus rhTNF-α-activated HUVECs *in vitro*

**Fig. 1:**
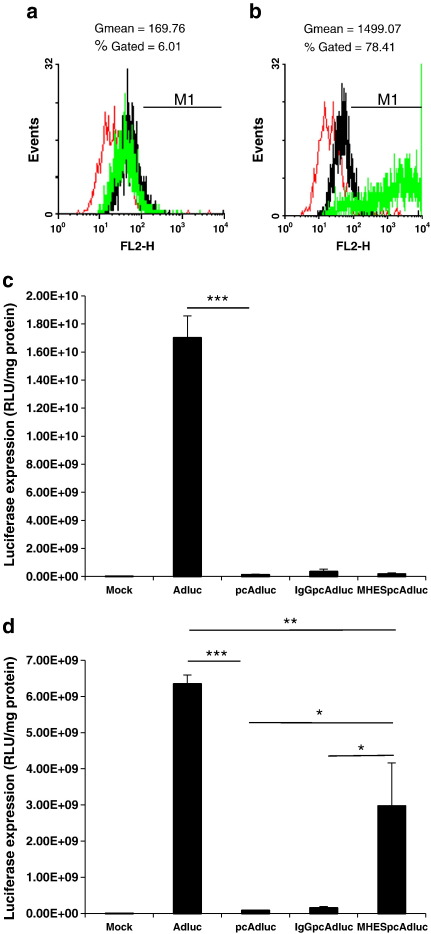
Correlation of MHES-retargeted virus transduction of HUVECs with TNF-α-mediated E-selectin up-regulation. (a, b) E-selectin expression in (a) non-rhTNF-α-activated and (b) rhTNF-α-activated HUVECs. (c, d) Transduction efficiency of MHES-retargeted polymer coated adenovirus (prepared by direct conjugation) in (c) E-selectin-negative and (d) E-selectin-positive HUVECs. Ad, unmodified adenovirus; pcAd, pHPMA-adenovirus; IgGpcAd, isotype control-pHPMA-adenovirus; and MHESpcAd, MHES-retargeted-pHPMA-adenovirus. Results are representative of at least three different experiments. Error bars are ± standard deviation. P values calculated using a one-way ANOVA (analysis of variance). **P < 0.01, ***P < 0.001.

In order to induce an activated phenotype in endothelial cells that would mimic the tumour microenvironment and induce E-selectin up-regulation, HUVECs were treated with rhTNF-α (100 ng/ml) for 4 h [12]. The E-selectin expression level in non-treated versus rhTNF-α-treated cells was determined by flow cytometry. Non-rhTNF-α-activated HUVECs do not express E-selectin (Fig. 1a) and were barely transduced by adenoviruses bearing mouse anti-human E-selectin monoclonal antibodies (MHESpcAdluc) (Fig. 1c). In contrast a 4-h rhTNF-α treatment up-regulated E-selectin expression (Fig. 1b), resulting in a significant increase in transduction efficiency of the E-selectin-targeted virus (Fig. 1d). MHESpcAdluc demonstrated 34.8-fold higher transgene levels compared with polymer coated virus and 18.3-fold higher infection efficiency in receptor-positive compared with receptor-negative cells. Retargeting virus with an isotype control antibody (IgGpcAdluc), did not restore viral infection in E-selectin-expressing cells, (Fig. 1d).

**Fig. 2:**
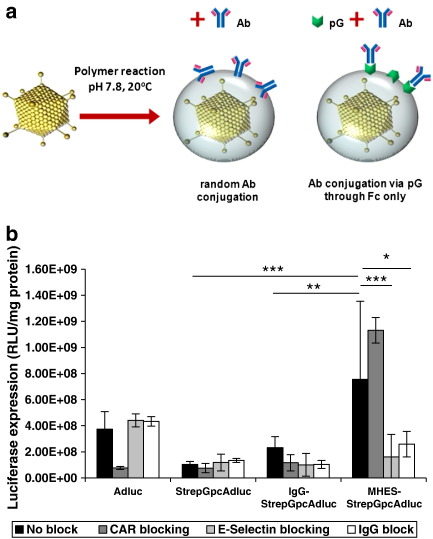
Streptococcal protein G as a platform system for targeting E-selectin *in vitro*. (a) Diagram illustrating concept of retargeting pcAdluc by linking antibodies via protein G. Polymer coated virus shown in grey. (b) Retargeting protein G-pHPMA-coated adenovirus via E-selectin in rhTNF-α-activated HUVECs. Ad, unmodified adenovirus; StrepGpcAd, protein G-pHPMA-adenovirus; IgG-StrepGpcAd, isotype control-protein G-pHPMA-adenovirus; and MHES-StrepGpcAd, MHES-retargeted-protein G-pHPMA-adenovirus. Results are representative of three different experiments. Error bars are ± standard deviation. P values calculated using a one-way ANOVA (analysis of variance). *P < 0.05, **P < 0.01, and ***P < 0.001.

Although MHESpcAdluc showed encouraging luciferase activity, the use of antibodies for direct conjugation to polymer coated viruses occurs randomly and is subject to variability between different antibodies depending on their availability of reactive amino groups (Fig. 2a). Hence, alternative strategies of ligand-conjugation were considered using Streptococcal protein G as a platform system for targeting receptors of interest.

### 3.2 Streptococcal protein G as a platform system for targeting E-selectin *in vitro*

In order to enable orientated linkage of Fc-bearing ligands including MHES monoclonal antibody, protein G was directly incorporated onto polymer coated virus (StrepGpcAdluc). The amount of protein G molecules associated with each virus was determined by dot blot analysis using a protein G standard curve (Supplementary Fig. 1). On average each virus particle (determined by pico green analysis) was associated with 169 attograms of protein G equivalent to 4700 (+/− 815) molecules of protein G. However by Western blot analysis we could not rule out the possibility that free protein G remained associated with the virus even after purification by caesium chloride centrifugation (data not shown); suggesting that this value is an over-estimate of the number of molecules covalently attached to the polymer. MHES or IgG antibodies, were then conjugated to StrepGpcAdluc to generate retargeted virus (MHES-StrepGpcAdluc, IgG-StrepGpcAdluc). As shown in Fig. 2b (black bars), StrepGpcAdluc generated minimal non-specific binding while MHES-StrepGpcAdluc demonstrated efficient endothelial transduction compared with both Adluc and IgG-StrepGpcAdluc. Blocking CAR knocks down Adluc infection by 4-fold with no effect on the transduction efficiency of MHES-StrepGpcAdluc (Fig. 2b dark grey bars). Furthermore, infection in the presence of competing free MHES monoclonal antibody (intended to outcompete binding for E-selectin), resulted in almost 5-fold less transgene expression in HUVECs transduced with the MHES-retargeted virus; while Adluc, protein G-modified and IgG-control viruses remained unaffected by E-selectin blockade. Free IgG monoclonal antibody also interfered with MHES-retargeted virus transduction, resulting in 2.9-fold decrease in infection efficiency. This observation suggests some possible limitations in retargeting using the protein G-platform system and highlights its potential susceptibility to competition from plasma IgGs.

### 3.3 Transduction efficiency of endothelial cells *ex vivo* in a human umbilical vein cord model

**Fig. 3:**
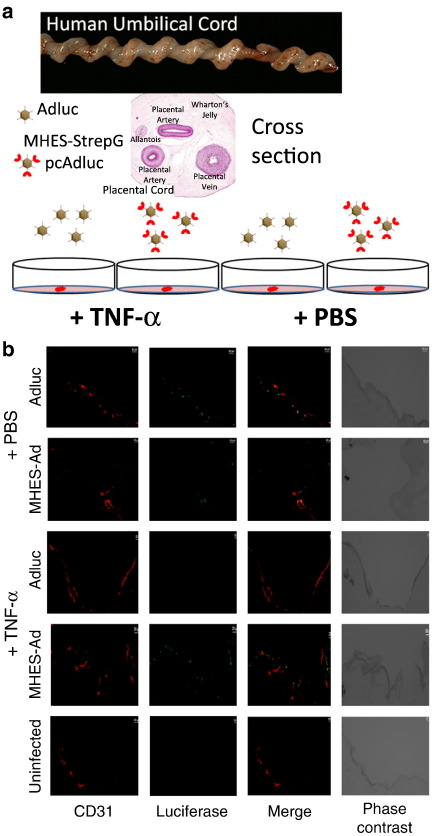
Transduction efficiency of MHES-StrepGpcAdluc in a human umbilical vein cord model *ex vivo.* (a) Diagram illustrating the handling procedure of human umbilical vein infection with Adluc and MHES-StrepGpcAdluc *ex vivo*. (b) Immunohistochemistry staining of human umbilical vein sections treated with PBS or rhTNF-α and infected with Adluc and MHES-StrepGpcAdluc. CD31 in red; Firefly luciferase in green; CD31/Firefly luciferase co-localisation is seen where the merged fluorescence is yellow. Scale bars represent 20 μm.

Because of the lack of cross species reactivity between mice and humans, the infection efficiency of the MHES-retargeted virus could not be tested *in vivo* in tumour-bearing mice. Instead, the ability of the virus to infect and transduce polarised human endothelial cells (within their correct architectural organisation), was tested *ex vivo* in a human umbilical vein cord model (Fig. 3a).

Immunohistochemical staining of umbilical cord sections infected (in the absence of activation by rhTNF-α) with Adluc, revealed traces of luciferase transgene expression (observed as green signals in Fig. 3b) which appeared to co-localise with CD31-stained endothelial cells (stained in red). In contrast infection with the MHES-retargeted virus gave very little transgene expression (Fig. 3b). However, when the cord was treated with rhTNF-α for 4 h prior to infection, a marked increase in transgene expression from MHES-StrepGpcAdluc was observed, likely reflecting up-regulation of E-selectin expression on the surface of polarised endothelial cells allowing their transduction. In contrast, luciferase transgene expression from the unmodified virus was abrogated after rhTNF-α treatment of the cord (Fig. 3b).

### 3.4 Protein G as a platform system for targeting viruses using alternative ligands *in vitro*

**Fig. 4:**
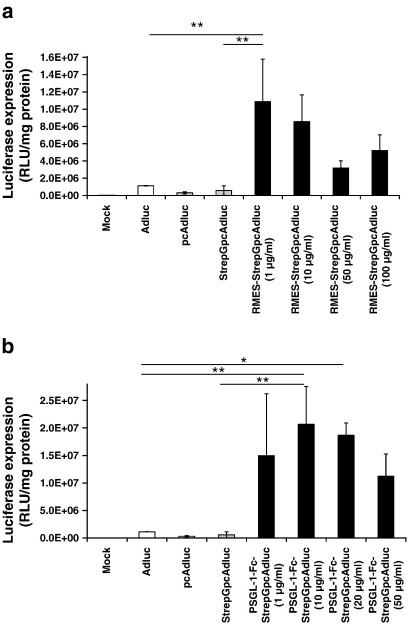
Retargeting protein G-pHPMA-coated adenovirus using alternative Fc-bearing ligands targeting murine endothelia. (a) Transduction of murine bEnd3 cells using StrepGpcAdluc retargeted via E-selectin with a rat anti-mouse E-selectin IgG2a monoclonal antibody (RMES mAb). (b) Transduction of murine bEnd3 cells using StrepGpcAdluc retargeted via P-selectin with a chimeric P-selectin Glycoprotein Ligand-1 (PSGL-1)-Fc fusion protein. In each case prior to infection cells were treated with recombinant murine-TNF-α (rmTNF-α, 120 ng/ml) for 5 h at 37 °C. Ad, unmodified adenovirus; pcAd, pHPMA-adenovirus; StrepGpcAd, protein G-pHPMA-adenovirus; RMES-StrepGpcAd, RMES retargeted-protein G-pHPMA-adenovirus; and PSGL-1-Fc-StrepGpcAd, PSGL-1 retargeted-protein G-pHPMA-adenovirus. Results are representative of at least three different experiments. Error bars are ± standard deviation. P values were calculated using a one-way ANOVA (analysis of variance). *P < 0.05, **P < 0.01.

One advantage of the protein G targeting system is flexibility in ligand choice allowing incorporation of any ligand bearing an appropriate Fc region. The ability of this system to target endothelial selectins, mostly E-selectin but also P-selectin expressed on endothelial cells, was tested using two alternative ligands. In order to target StrepGpcAdluc to murine vasculature associated with human tumour xenografts, a rat anti-mouse E-selectin IgG2a monoclonal antibody (RMES mAb) and a chimeric P-selectin Glycoprotein Ligand-1 (PSGL-1)-Fc fusion protein were tested as alternative targeting ligands. The incorporation of RMES mAb resulted in an enhancement in the transduction efficiency of murine bEnd3 cells, usually very poorly transduced by adenovirus, with an optimal ligand concentration of 1 μg/ml (Fig. 4a). At this concentration RMES-StrepGpcAdluc generated 10-fold higher transgene expression compared to the non-modified virus. Retargeting the virus with PSGL-1-Fc also resulted in improved transduction of murine endothelium, in a ligand concentration dependent manner, which at optimal concentration (10 μg/ml) generated 18.4-fold greater endothelial transduction compared with unmodified virus and 35.4-fold higher transgene expression than StrepGpcAdluc (Fig. 4b). Based on performance criteria *in vitro*, the PSGL-1-Fc-targeting system was taken forward for study *in vivo*.

### 3.5 Targeting pHPMA-adenovirus to selectin-expressing endothelium *in vivo*

**Fig. 5:**
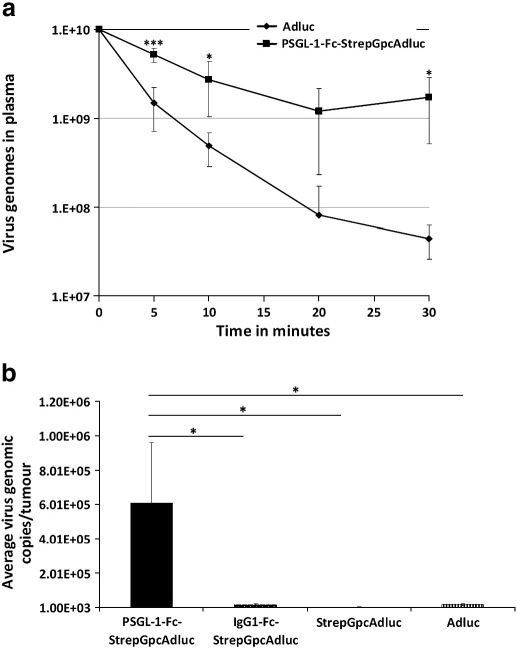
Pharmacokinetics of PSGL-1-Fc retargeted virus. (a) Blood circulation profile of PSGL-1-Fc-StrepGpcAd (squares) and Adluc (diamonds) *in vivo* following intravenous administration of 1e10 virus particles. (b) Number of virus genomic copies recovered from HepG2 xenograft tumours following administration of Adluc, StrepGpcAdluc, IgG1-StrepGpcAdluc or PSGL-1-Fc-StrepGpcAdluc. P values were calculated using a one-way ANOVA (analysis of variance). *P < 0.05.

Intravenous injections of viruses in tumour-bearing mice revealed marked differences in blood circulation profiles between Adluc and the retargeted virus (PSGL-1-Fc-StrepGpcAdluc). While Adluc experienced a rapid clearance from the bloodstream, measured by real time (quantitative) PCR, falling to 1% of the input dose remaining in the plasma after 20 min, compatible with the first pass hepatic clearance mechanism; the PSGL-1-retargeted virus showed extended circulation kinetics, with approximately 17.1% of the input dose still in the bloodstream after 30 min (Fig. 5a). This is approximately 38 times higher than the unmodified virus. This enhanced circulation property is attributed to the pHPMA polymer used to coat the virus, prolonging its survival in the bloodstream as previously demonstrated by Green et al*.* [27]. This extension in plasma circulation time thus provides a better platform for efficient targeting of distant tumour sites.

The ability of the PSGL-1-retargeted virus to reach a distant subcutaneous tumour following systemic administration was subsequently investigated in a separate study in which PSGL-1-Fc-StrepGpcAd was directly compared with non-modified virus (Adluc) and two control modified viruses (StrepGpcAd and IgG1-Fc-StrepGpcAd). Following intravenous administration of 1 × 10^10^ virus particles/mouse, PSGL-1-Fc-StrepGpcAdluc efficiently accumulated in HepG2 human tumour xenografts implanted subcutaneously into CD_1_ nude mice, with an overall ratio of 345.5-, 36- and 43.7-fold higher virus genomic copies compared with StrepGpcAdluc, Adluc and the IgG1-Fc-StrepG-modified control virus, respectively (Fig. 5b).

### 3.6 Efficiency of the PSGL-1-Fc-retargeted virus to infect endothelial cells in tumour xenografts following systemic administration

**Fig. 6:**
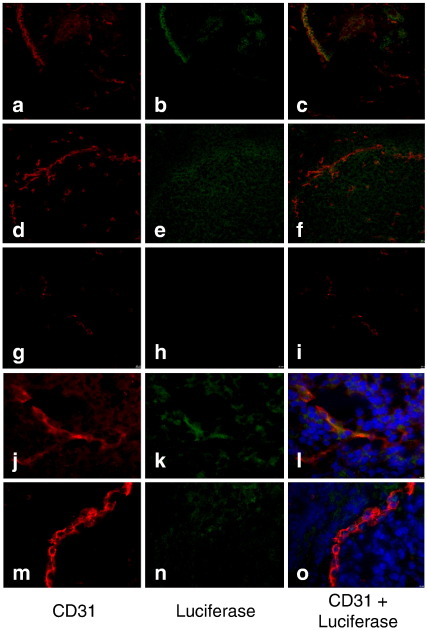
Targeting endothelial cells in human tumour xenografts by systemic vector administration. Cryostat sections (10 μm) of HepG2 tumour xenografts from mice injected with Adluc or PSGL-1-Fc-StrepGpcAdluc. Photomicrographs were taken using a confocal microscope (Nikon Optiphot-2 upright microscope). (a–c) 20× and (j–l) 60× PSGL-1-Fc-StrepGpcAdluc-infected tumour sections; (d–f) 20× and (m–o) 60× Adluc-infected tumour sections; (g–i) 20× PBS-treated tumour sections. CD31 in red; Firefly luciferase in green; CD31/Firefly luciferase co-localisation (orange/yellow).

The previous findings have shown that the PSGL-1-retargeted virus can accumulate efficiently into tumours following systemic administration. However these quantification studies do not reveal which cell population within the tumour tissue has been infected. Since the retargeted virus was designed to infect endothelial cells, it was important to assess whether the expressed transgene co-localised with the endothelial cells of HepG2 tumour xenografts. Tumour sections from PSGL-1-Fc-retargeted virus and Adluc-treated mice were double-stained for CD31 (an endothelial marker) and firefly luciferase (the expressed transgene). Different patterns of staining were observed in tumours from animals treated with the PSGL-1-retargeted virus (Fig. 6a-c, j–l) compared with those treated with the non-modified virus (Fig. 6d–f, m–o). While a more endothelial-localised luciferase staining (shown in green) was observed in PSGL-1-Fc-StrepGpcAdluc-infected tumour sections, a disperse staining in close proximity but not within blood vessels was seen in Adluc-infected tumour sections. A co-localisation of luciferase expression (green signal) with CD31-positive tumour-associated endothelium (red signal) was confirmed if a yellowish signal could be visualised after merging individual luciferase-positive and CD31-positive pictures. Tumour sections from mice treated with PBS were used as a negative control to account for any unspecific binding from the antibody used to stain luciferase in tumour samples transduced by Adluc and the retargeted virus. The results clearly show that PSGL-1-Fc-StrepGpcAdluc and Adluc are not infecting the same cell populations within the tumour tissue and that the retargeted virus is indeed specifically infecting endothelial cells.

## 4 Discussion

Adenoviral vectors do not generally perform well following intravenous delivery because they are rapidly inactivated and cleared by the innate and adaptive immune system. Our group has previously shown that stealthing adenovirus particles with multivalent reactive copolymers based on poly-[N-(2-hydroxypropyl)methacrylamide] (pHPMA) protects virus surface epitopes from interaction with the environment [23] and gives an impressive extension in plasma circulation kinetics following intravenous delivery [27]. The tropism-ablated polymer coated virus can then be retargeted with various molecules including growth factors [23,28–30], monoclonal antibodies [24] and peptides [22] to infect cells via endosome-internalising receptors of interest.

Endothelial selectins represent an attractive target, given their physiological roles in binding circulating blood cells under shear blood flow conditions, and their pattern of selective expression at sites of inflammation such as in tumours. *In vitro* a 4-h activation of HUVECs with rhTNF-α is sufficient to induce a substantial (8-fold increase in GMean value) up-regulation in the E-selectin level. This enables ligand-specific transduction of E-selectin-expressing endothelial cells, following infection with the MHES-retargeted virus.

While the linkage of E-selectin antibodies to the surface of adenovirus has previously been reported for targeting activated endothelial cells, previous strategies have employed PEG to mediate attachment [12]. Ogawara et al. used bifunctional PEG containing N-hydroxysuccinimide ester (NHS) and vinyl sulfone (VS) groups at each end of the molecule, to react (via NHS esters) with primary amino groups on the surface of the virus preventing fibre/CAR attachment, while remaining VS groups were available to bind targeting antibodies. The use of multivalent pHPMA based copolymers, which are able to bind to the virus surface by multiple linkages [23], provides not only a platform to prevent CAR/intergrin mediated uptake and attachment of targeting antibodies but can also improve shielding against components within the blood [31]. Avoidance of neutralising antibodies, coagulation factors and complement is a prerequisite for systemic delivery routes accessing tumours via the vasculature.

The use of antibodies for direct conjugation to polymer coated viruses, remains however problematic. This strategy is subject to variability between different antibody molecules depending on their content of reactive amino groups, leading to a mixture of orientations sometimes with exposed Fc regions, which might resemble immune complexes. Hence, strategies to minimise Fc exposure were deemed important and alternative approaches of orientated ligand-conjugation were investigated based on adaptor proteins.

Following the reported success of using *S. aureus* protein A to correctly orientate antibody based ligands [25], we tested the use of protein A to attach MHES to polymer coated Ad. Transduction of TNF-α stimulated HUVECs resulted in a 30-fold increase in transgene expression from MHES-StaphApcAdluc compared with Adluc (data not shown). However the use of an isotype control antibody also substantially increased transduction of the endothelial cells compared to the unmodified virus (data not shown). Even without addition of targeting antibody, the presence of the *S. aureus* protein A increased transgene expression 4-fold over Adluc (data not shown). Given the high degree of non-specific binding engendered by protein A in this configuration, attention was focussed on an alternative mechanism of attachment namely *Streptococcal aureus* protein G.

Using *in vitro* studies we have validated the concept of using protein G-modified virus to allow spontaneous binding of monoclonal antibodies against E-selectin, leading to receptor-mediated infection. Importantly this method unlike protein A did not result in substantial non-specific binding and transduction of target cells (Fig. 1c and d, compare pcAdluc and IgGpcAdluc). This system could clearly be easily adapted for linkage of any Fc-containing retargeting ligand. In this manner we believe that protein G modification will provide a useful tool to screen candidate cell surface receptors for targeting, by displaying correctly oriented antibodies on the surface of the polymer coated virus.

In an *ex vivo* model of human umbilical vein cord transduced with Adluc or MHES-StrepGpcAdluc following treatment with PBS or rhTNF-α, the ability of the retargeted virus to infect a polarised layer of endothelial cells was demonstrated. Following stimulation of the cells with rhTNF-α, the non-modified (Ad) virus failed to show transduction, while the E-selectin-targeted virus gave a high frequency of transduced cells. This is in agreement with rhTNF-α-induced over expression of E-selectin *in vitro, ex vivo* and *in vivo* [32–34]. The inability of unmodified Ad to infect endothelial cells in the presence of rhTNF-α might reflect down-regulation of viral receptors. While rhTNF-α increases the expression of E-selectin, P-selectin, ICAM-1, and vascular cell adhesion molecule (VCAM-1) on endothelial cells [35], it can also change the distribution of some surface proteins away from sites of inter-endothelial cell contact [36]. It can also down-regulate cell surface expression of some proteins such as: platelet endothelial cell adhesion molecule-1 (PECAM-1) [37,38] as well as αvβ3 integrin [39] and CAR [40]; the pivotal receptors for Ad binding and internalisation [41,42].

This system was also tested *in vivo*, where a protein G-modified virus retargeted with a chimeric P-selectin Glycoprotein Ligand-1 (PSGL-1)-Fc fusion protein was injected i.v. to target tumour-associated vasculature in tumour-bearing mice. The retargeted virus showed endothelial cell transduction and a better tumour accumulation with an average of 36 fold higher virus uptake compared with unmodified virus.

The ability of free antibodies to outcompete the retargeting ligand for the protein G-modified virus (Fig. 2b) must be taken into consideration in the design of extended duration experiments, although it appears to have only limited consequences for *in vivo* targeting studies where virus kinetics are generally short.

These findings highlight the possibility to deliver genes selectively to tumour-associated vasculature providing an alternative to direct tumour cell targeting. Endothelial targeting bypasses the need for extravasation which can limit targeting delivery.

The following are the supplementary materials related to this article.

Supplementary Fig. 1

## Conflict of interest

K.D.F. and L.W.S are directors of Hybrid BioSystems Ltd., which owns the intellectual property on adenovirus coating using multivalent reactive pHPMA polymers. No competing financial interests exist for H.B. and M.S.
