# Supplementary material for: Targeting adenovirus gene delivery to activated tumour-associated vasculature via endothelial selectins
Source: J Control Release. 2011 Mar 10;150(2):196–203. doi: 10.1016/j.jconrel.2010.10.011 (PMC3071491; doi:10.1016/j.jconrel.2010.10.011)
Supplement: Supplementary file 1 — Supplementary Fig. 1 [file mmc1.ppt]

## Slide 1
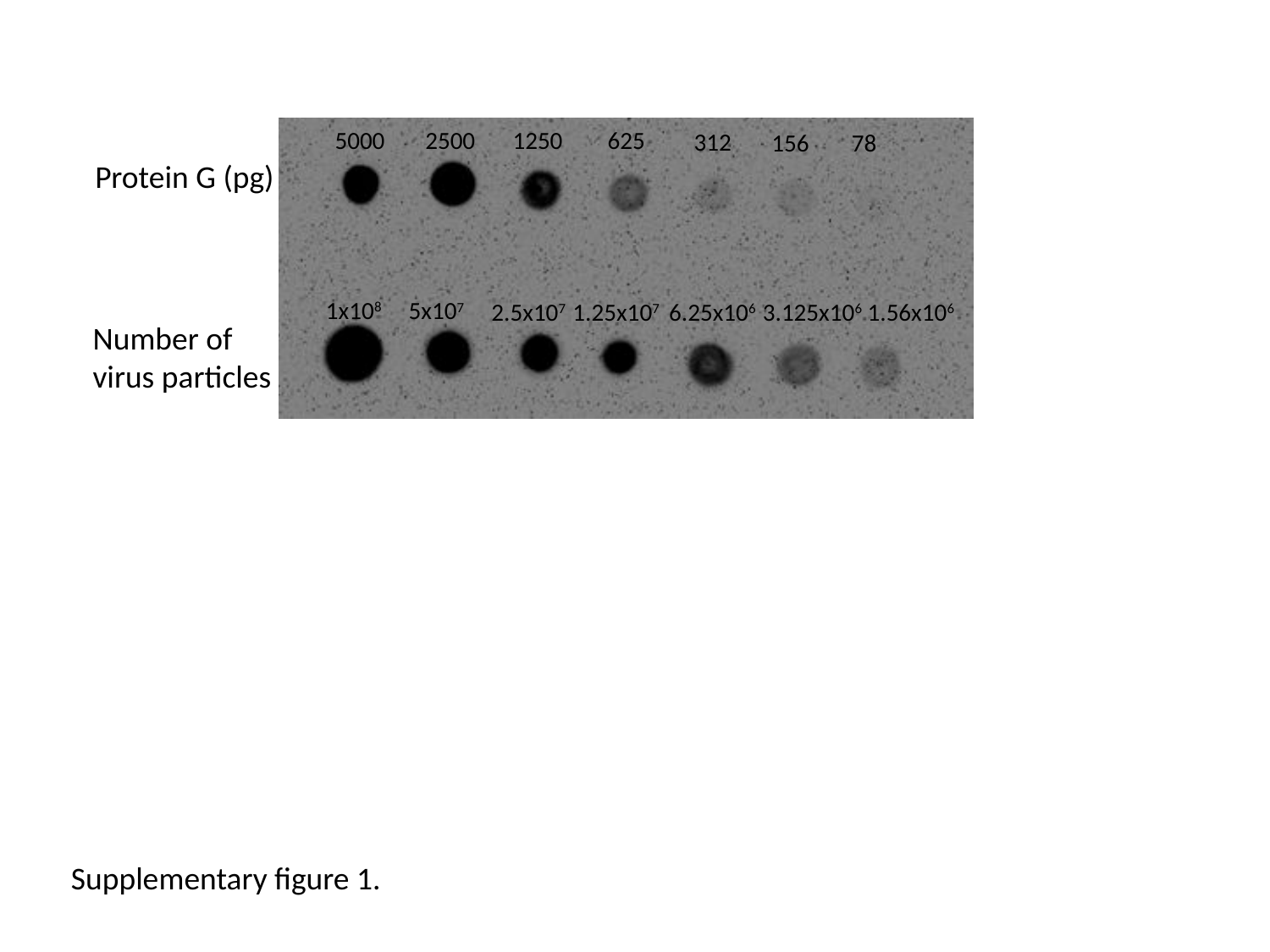

5000
2500
1250
625
312
156
78
Protein G (pg)
1x10^8^
5x10^7^
2.5x10^7^
1.25x10^7^
6.25x10^6^
3.125x10^6^
1.56x10^6^
Number of virus particles
Supplementary figure 1.

## Slide 2
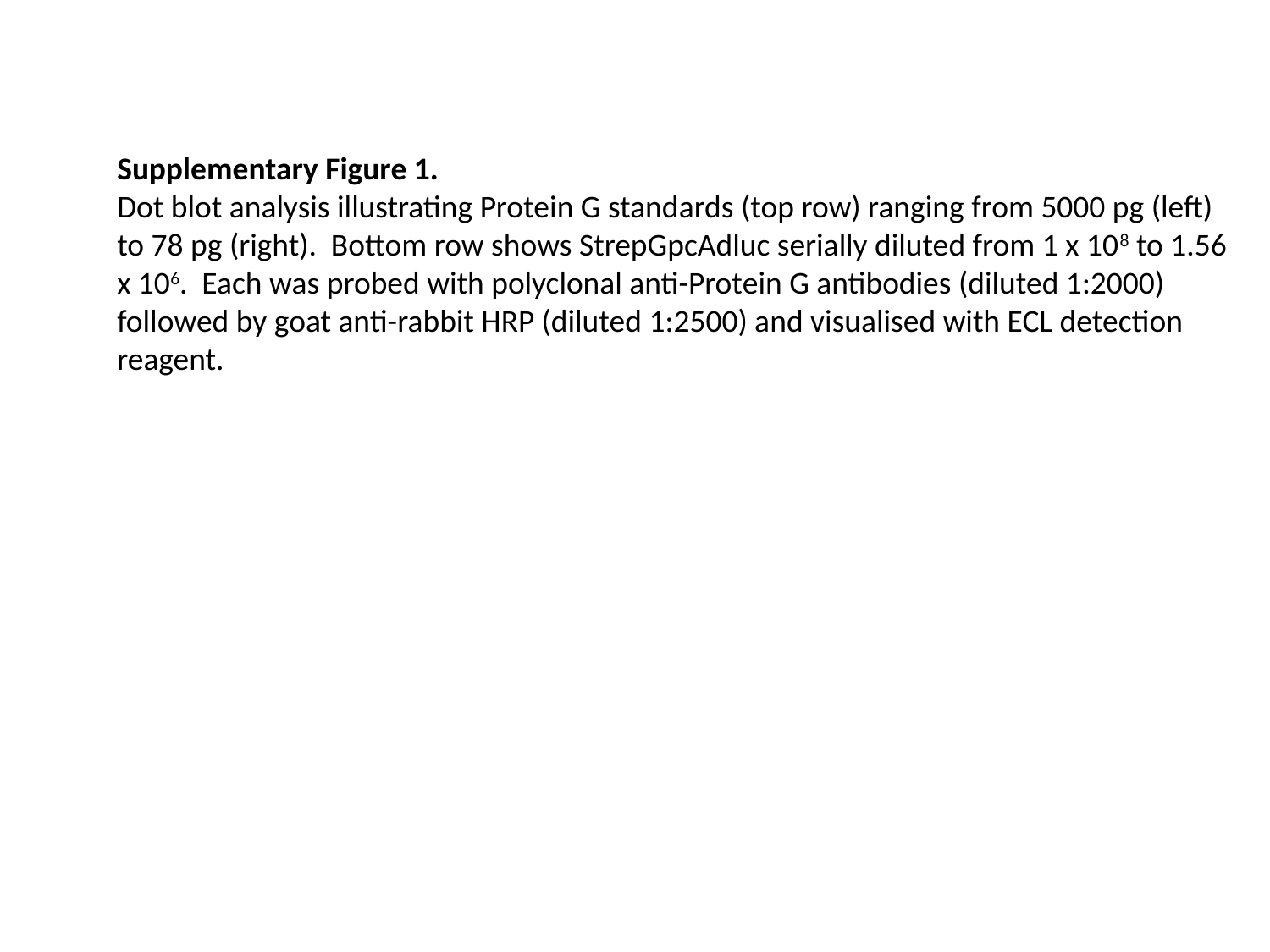

Supplementary Figure 1.
Dot blot analysis illustrating Protein G standards (top row) ranging from 5000 pg (left) to 78 pg (right). Bottom row shows StrepGpcAdluc serially diluted from 1 x 10^8^ to 1.56 x 10^6^. Each was probed with polyclonal anti-Protein G antibodies (diluted 1:2000) followed by goat anti-rabbit HRP (diluted 1:2500) and visualised with ECL detection reagent.
